# Bioactive, Functional, and Technological Properties of Gluten-Free Pasta Enriched with Mango (*Mangifera indica* L.) Leaf Powder

**DOI:** 10.3390/foods14234006

**Published:** 2025-11-22

**Authors:** Génica Lawrence, Ewa Pejcz, Ingrid Marchaux, Agata Wojciechowicz-Budzisz, Remigiusz Olędzki, Guylène Aurore, Joanna Harasym

**Affiliations:** 1 Campus de Fouillole, Université des Antilles, COVACHIM-M2E (EA 3592), UFR SEN, F-97110 Pointe-à-Pitre, France; genica.lawrence@univ-antilles.fr (G.L.); imarchaux@gmail.com (I.M.); guylene.aurore@univ-antilles.fr (G.A.); 2 Department of Biotechnology and Food Analysis, Wroclaw University of Economics and Business, Komandorska 118/120, 53-345 Wroclaw, Poland; agata.wojciechowicz-budzisz@ue.wroc.pl (A.W.-B.); remigiusz.oledzki@ue.wroc.pl (R.O.); joanna.harasym@ue.wroc.pl (J.H.); 3 Adaptive Food Systems Accelerator-Science Centre, Wroclaw University of Economics and Business, Komandorska 118/120, 53-345 Wroclaw, Poland

**Keywords:** mango leaves, gluten-free pasta, *Mangifera indica*, antioxidant activity, techno-functional properties, Nam Dok Mai

## Abstract

Mango (*Mangifera indica* L.) leaves represent an underutilised plant resource rich in phenolic compounds and dietary fibre with potential applications in functional foods. This study evaluated the effects of incorporating mango leaf powder (MLP) from five cultivars (Nam Dok Maï, Julie, DLO, Irwin, and Keitt) into gluten-free chestnut flour–based pasta, focusing on techno-functional, rheological, and bioactive properties. Among the formulations tested, Nam Dok Mai and Julie cultivars at 10% substitution demonstrated the most favourable pasting behaviour and were selected for spaghetti production. Both variants exhibited acceptable texture and cooking quality; however, Nam Dok Mai spaghetti showed superior colour stability after cooking and storage. Although Julie exhibited higher setback viscosity, which indicates greater starch retrogradation potential, cutting force measurements showed no significant differences between cultivars. Antioxidant analyses (DPPH, ABTS, FRAP) and total polyphenol content revealed that Nam Dok Mai retained a higher level of bioactive compounds following thermal processing, particularly in aqueous extracts. These findings indicate that mango leaf incorporation can enhance the functional value of gluten-free pasta without compromising technological quality, and that Nam Dok Mai represents the most promising cultivar for such applications.

## 1. Introduction

Consumer demand is increasingly directed towards foods that combine health benefits with environmental sustainability [1]. Gluten-free and functional products enriched with bioactive compounds from local resources represent a rapidly growing segment of the food market [2,3]. The gluten-free diet is indispensable for individuals suffering from celiac disease or gluten intolerance, but it has also gained popularity among health-conscious consumers [4]. Despite the expanding demand, gluten-free pasta often presents nutritional and technological limitations, such as reduced protein content, weaker cooking quality, and less desirable sensory properties compared to traditional wheat pasta [5,6]. Addressing these challenges requires the integration of nutrient-rich and bioactive ingredients capable of enhancing both the health-promoting potential and technological performance of gluten-free products.

Pasta is one of the most widely consumed staple foods worldwide, valued for its nutritional profile, shelf stability, and versatility [7]. Due to its global relevance, pasta has often been used as a carrier of functional ingredients, including dietary fibers, proteins, and phytochemical-rich plant materials [8,9,10]. Such enrichment approaches aim to improve their nutritional quality and deliver additional health benefits, while ensuring consumer acceptability. However, gluten-free pasta remains particularly challenging, as the absence of gluten reduces dough elasticity, firmness, and overall textural properties [6]. This has driven efforts to identify alternative flours and functional plant-based ingredients that can restore technological quality while adding nutritional value.

Functional enrichment of pasta with bioactive compounds has become a growing research focus in food science. Numerous plant-based sources, such as green tea extract, grape seed flour, spirulina, and berry powders, have been studied for their potential to enhance antioxidant activity, polyphenol content, and overall health-promoting value of pasta [11,12,13]. These studies highlight the dual role of such enrichment: not only increasing nutritional and bioactive properties, but also influencing pasta color, texture, and consumer acceptance. However, plant biomass addition may negatively affect sensory quality if not properly balanced, underlining the importance of identifying suitable ingredients that provide both functional benefits and technological compatibility.

Among potential raw materials, mango (*Mangifera indica* L.) leaves stand out as an underutilised but promising source of nutrients and phytochemicals. Traditionally used in herbal medicine and functional beverages, mango leaves are rich in phenolic compounds such as mangiferin, quercetin, flavonoids, and benzophenones, along with minerals and vitamins [14,15]. These compounds are associated with antioxidant, anti-inflammatory, antimicrobial, and metabolic regulatory activities [14]. Recent studies have emphasized their potential in preventing oxidative stress and modulating chronic disease risk factors [15]. Despite this high bioactive potential, mango leaves remain largely unexplored in food formulation, particularly in staple gluten-free products such as pasta. Their valorization would not only introduce health-promoting compounds into a widely consumed food matrix but also contribute to the sustainable utilization of tropical plant resources.

In Guadeloupe, a French overseas territory in the Caribbean where the raw material was obtained, mango cultivation is well-established, with over 200 cultivars grown locally [16]. Mango trees, including leaves, represent an abundant and renewable local resource in tropical and subtropical regions worldwide [14,15]. The valorization of mango leaves as a functional ingredient represents an opportunity to develop locally sourced, sustainable ingredients, particularly relevant for tropical mango-producing regions, including the Caribbean, Southeast Asia, India, and parts of Africa and Latin America.

Previous research on pasta fortification has mainly focused on fibers, protein concentrates, cereal brans, or aromatic plant extracts [8,9,10]. There are no studies investigating the direct incorporation of tropical fruit tree leaves, such as mango leaves, into pasta formulations. Considering their phytochemical richness and functional potential, mango leaves could serve as an innovative and sustainable ingredient for gluten-free pasta production. This study aimed to evaluate the effects of mango leaf powder incorporation on the functional, bioactive, rheological, and technological properties of gluten-free pasta and its precursor flour blends. It was hypothesised that mango leaf addition would enhance the phenolic content and antioxidant capacity while maintaining acceptable physicochemical and structural quality of the final product.

## 2. Materials and Methods

### 2.1. Materials and Preparation of Mango Leaf Powders and Flour Blends

Mango leaves were collected, washed, and dried at 42 °C and 30% relative humidity for 72 h in a food dryer (Tauro Essiccatori Biosec Pro 2200 W). After drying, the leaves were ground to a fine powder using an electric grinder (Taurus Aromatic 150 W). The resulting powders were immediately vacuum-packed and stored at room temperature (23 ± 1 °C) until use (maximum 48 h). These procedures were identical to those described by Lawrence et al. (2025) [16], where detailed particle size characterization is provided. Several mango cultivars were used, including Nam Dok Maï, Julie, DLO, Irwin, and Keitt. The process involved leaf collection, drying, and fine milling to obtain homogeneous powders suitable for blending with chestnut flour.

Composite blends were prepared by partially substituting chestnut flour (CF) with mango leaf powder at two incorporation levels (10% and 20% *w*/*w*). These blends were used for the determination of techno-functional parameters, total polyphenol content (TPC), antioxidant activity (DPPH, ABTS, FRAP), and reducing sugar content. All measurements were performed in triplicate on freshly prepared blends.

### 2.2. Techno-Functional Characterization of Blends

The techno-functional properties of the mango leaf–chestnut flour blends were assessed through a series of standardized analytical procedures evaluating water-holding capacity (WHC), water absorption capacity (WAC), oil absorption capacity (OAC), hydrophilic/lipophilic index (HLI), water absorption index (WAI), water solubility index (WSI), and swelling power (SP). All measurements were carried out in triplicate.

The determination of WHC and WAC followed the protocol described by Vingadassalon et al. (2024) [17] with minor modifications. For WHC, 3 g of each blend sample was placed in a pre-weighed centrifuge tube containing 30 mL of distilled water. The dispersions were left to stand at room temperature for 24 h to allow hydration under gravitational conditions. The supernatants were then carefully decanted, and the hydrated sediments were weighed. WHC was expressed as grams of water retained per gram of dry sample (g/g dm).

For WAC, the same amount of sample (3 g) was mixed with 30 mL of distilled water, vortexed for 30 s, and centrifuged at 3000× *g* for 10 min (Thermo Fisher Scientific, Waltham, MA, USA). The supernatant was discarded, and the residue was dried at 50 °C until constant weight in a laboratory oven (Vindon Scientific, Rochdale, UK). WAC was calculated as the total amount of water absorbed and retained (g/g dm) after centrifugation and drying [17].

OAC was determined under the same operational conditions, except that 30 mL of rapeseed oil was used instead of water. After vortexing and centrifugation, the unbound oil was decanted, and the tubes were placed in a drying oven at 50 °C for 25 min to evaporate residual oil. The results were expressed as grams of oil retained per gram of dry sample (g/g dm). The hydrophilic/lipophilic index (HLI) was calculated as the ratio of WAC to OAC, indicating the relative affinity of the blends toward polar and non-polar media [17].

The water absorption index (WAI), water solubility index (WSI), and swelling power (SP) were measured following the procedure of Harasym et al. (2020) [18], with slight modifications. Approximately 3 g of each blend was suspended in 30 mL of distilled water in pre-weighed centrifuge tubes and heated in a water bath at 90 °C for 10 min (MLL147, AJL Electronics, Kraków, Poland). After cooling to room temperature, the tubes were centrifuged at 3000× *g* for 10 min. The supernatant was carefully decanted into pre-weighed Petri dishes and dried at 110 °C for 24 h (SML, Zalmed, Łomianki, Poland) to determine the mass of soluble solids. The sediment was weighed to calculate WAI (g water/g dm), while WSI was expressed as grams of soluble solids per 100 g of dry sample (g/100 g dm). Swelling power (SP) was determined as the ratio of the hydrated sediment weight to the dry matter content (g/g dm) [18].

### 2.3. Determination of Total Polyphenol Content and Antioxidant Capacity

The extracts for the determination of total polyphenol content (TPC) and antioxidant capacity were prepared using both aqueous and hydroalcoholic (80% ethanol, *v*/*v*) solvents. Each extraction solvent was acidified with 1% HCl to enhance the breakdown of plant cell walls and improve the release of phenolic compounds [19]. Exactly 1 g of the blend sample was suspended in 10 mL of solvent and mixed using a laboratory orbital shaker (WU4, Premed, Marki, Poland) for 1 h at room temperature. The mixtures were then centrifuged at 3500× *g* for 10 min (MPW-350, MPW, Warsaw, Poland), and the supernatants were collected for subsequent analyses.

Total polyphenol content (TPC) was determined by the Folin–Ciocalteu colorimetric method [20]. A 20 μL aliquot of extract was mixed with 100 μL of Folin–Ciocalteu reagent and 1.58 mL of distilled water. After 5–8 min of incubation at room temperature, 300 μL of saturated Na_2_CO_3_ solution was added. The mixture was incubated for 30 min at 38 °C (MLL147, AJL Electronics, Kraków, Poland), and absorbance was measured at 765 nm using a UV–Vis spectrophotometer (SEMCO S91E, Sosnowiec, Poland). The results were expressed as mg gallic acid equivalents (GAEs) per g dry matter (DM), based on a calibration curve.

The antioxidant activity of the extracts was evaluated by DPPH, ABTS, and FRAP assays, each performed in triplicate.

In the DPPH radical scavenging assay, 34.5 μL of extract was added to 1 mL of 0.1 mM DPPH methanolic solution (absorbance 0.9 ± 0.1 at 517 nm). After 20 min of incubation at room temperature in the dark, absorbance was measured at 517 nm [21]. The results were expressed as mg Trolox equivalents (TEs) per g DM.

The ABTS•^+^ radical cation decolorization assay was carried out according to the method of Sridhar et al. [22], with slight modifications. Briefly, 20 μL of extract was added to 1 mL of freshly prepared ABTS•^+^ working solution diluted in phosphate buffer (pH 7.4). The decrease in absorbance was recorded at 734 nm after 10 s of mixing. Results were expressed as mg TE per g DM.

The ferric reducing antioxidant power (FRAP) assay was performed as described by Re et al. [23], with modifications. One millilitre of freshly prepared FRAP reagent was mixed with 0.0345 mL of extract. After incubation at 36 °C for 15–20 min, absorbance was read at 593 nm using a spectrophotometer (SEMCO S91 E, Warsaw, Poland). The reducing capacity was calculated from a ferrous sulfate (FeSO_4_·7H_2_O) calibration curve and expressed as mg Fe^2+^ equivalents per g DM.

### 2.4. Determination of Reducing Sugars

The content of reducing sugars in the aqueous and ethanolic extracts was determined using the dinitrosalicylic acid (DNS) colorimetric method [24]. A 0.5 mL aliquot of each extract was mixed with 0.25 mL of DNS reagent (1% 3,5-dinitrosalicylic acid in 0.4 M NaOH) and heated in a boiling water bath for 5 min. After cooling to 50–60 °C, the reaction mixture was diluted with 3 mL of distilled water. Absorbance was measured at 530 nm using a UV–Vis spectrophotometer (SEMCO S91E, Sosnowiec, Poland). Glucose was used to construct the calibration curve, and results were expressed as mg glucose equivalents (GEs) per g dry matter.

### 2.5. Rheological (Pasting) Properties of Selected Blends

Based on the results of functional and compositional analyses, the following blends were selected for rheological evaluation: CF (chestnut flour control) and the mango leaf–chestnut flour blends with mixing ratios of Nam Dok Mai 10/90, Nam Dok Mai 20/80, Irwin 10/90, Irwin 20/80, Julie 10/90, and Julie 20/80. These samples exhibited the most promising techno-functional characteristics and were therefore subjected to pasting property assessment.

The rheological behavior of the selected blends was determined using a Rapid Visco Analyzer (RVA 4500, Perten Instruments, Macquarie Park, Australia) according to the method described by Masiala et al. (2024) [25], with slight adjustments. For each measurement, 3.5 ± 0.01 g of sample (adjusted to 14% moisture content) was combined with 25 ± 0.01 g of distilled water in an aluminum canister. The total water addition was corrected to achieve a uniform solids concentration across samples. The mixture was homogenized with a plastic paddle prior to analysis to prevent lump formation.

A standard temperature–time program was applied, generating a viscosity curve over a total runtime of approximately 16 min. The procedure consisted of the following phases: an initial hold at 50 °C for 1 min, heating to 95 °C at a rate of 12.2 °C/min, a holding phase at 95 °C for 2 min, cooling back to 50 °C at 11.8 °C/min, and a final hold at 50 °C for 5 min. Paddle rotation was maintained at 960 rpm for the first 10 s, then reduced to 160 rpm. The parameters recorded from the pasting curve included peak viscosity, trough viscosity, breakdown, final viscosity, setback, and pasting temperature. Each measurement was performed in duplicate to ensure reproducibility.

### 2.6. Preparation of Gluten-Free Pasta

Gluten-free spaghetti was prepared using Nam Dok Maï and Julie mango leaf powders (MLs), selected based on their functional and rheological performance in the preliminary blend screening. The formulations consisted of chestnut flour (CF) as the base ingredient, tapioca starch (TS) to improve firmness and elasticity, eggs, olive oil, and water to ensure a cohesive dough. The proportion of mango leaf powder was set at 10% substitution (*w*/*w*) relative to CF, representing the optimal incorporation level identified during preliminary trials).

The formulations consisted of 450 g chestnut flour (CF), 150 g tapioca starch (TS), 60 g mango leaf powder (10% *w*/*w* substitution relative to CF), 3 whole eggs (approximately 150 g), 30 mL olive oil, and 180–200 mL water (adjusted to achieve optimal dough consistency). All dry ingredients were first homogenized, followed by the gradual addition of eggs, oil, and water. The mixture was blended for 2 min 30 s at low speed (force 1) and then allowed to rest for 30 min at room temperature (20 ± 1 °C) before extrusion. The dough was extruded into spaghetti form, and the fresh pasta strands were dried in an oven at 120 °C for 10 min. This rapid, high-temperature drying method represents a departure from traditional low-temperature pasta drying (typically 40–60 °C for several hours) and was deliberately selected based on preliminary optimization trials. The rationale was to minimize the total exposure time to elevated temperature, thereby potentially reducing cumulative thermal degradation of phenolic compounds through rapid moisture removal and enzyme inactivation. High temperature and short-term were related to stickiness of pasta, and the aim was to close the surface as soon as possible, to prevent leakage. After 10 min, the pasta was left for further drying at 40 °C for 6 h.

The prepared spaghetti samples were subjected to further analyses, including color, texture, and antioxidant properties, both immediately after preparation (fresh) and after 3 days of refrigerated storage at 4 °C.

### 2.7. Evaluation of Pasta Quality

#### 2.7.1. Color Analysis

Color measurements of the spaghetti samples were performed before cooking, after cooking, and after 3 days of storage at 4 °C to assess pigment stability and processing-induced color changes. A Konica Minolta CR-310 colorimeter (Ramsey, NJ, USA) equipped with a D65 light source was used. Prior to measurement, the device was calibrated with a white and black reference tile. The L* (lightness), a* (red-green), and b* (yellow-blue) values were recorded as the mean of readings collected from three distinct surface regions of each sample. Spaghetti samples were placed on a white ceramic plate in parallel strands (minimum 10 strands per measurement area) to create a uniform surface. The colorimeter probe was positioned perpendicular to the pasta surface with direct contact. Three distinct surface regions per sample were measured, with each region representing a different location along the spaghetti length. For cooked samples, pasta was drained, gently blotted with paper towels to remove surface water, and immediately measured. Stored samples were brought to room temperature (20 ± 1 °C) prior to measurement. No grinding or homogenization was performed to preserve the actual appearance of the product as consumed.

#### 2.7.2. Texture Analysis

The cutting force of cooked spaghetti samples, analyzed both immediately after cooking and after storage, was determined using a Texturemeter FC200STAV50 (AXIS Ltd., Gdańsk, Poland). The maximum force (N) required to cut through the strand was recorded as an indicator of firmness. Measurements were conducted in triplicate.

#### 2.7.3. Determination of Total Polyphenols and Antioxidant Activity in Pasta

To assess the retention of bioactive compounds after processing, ethanolic and aqueous extracts of the cooked spaghetti were prepared. The total polyphenol content (TPC) and antioxidant activities (DPPH, ABTS, and FRAP assays) were determined following the same analytical protocols as applied for the mango leaf–chestnut flour blends. All measurements were conducted in triplicate using freshly cooked pasta and after refrigerated storage (3 days at 4 °C).

### 2.8. Statistical Analysis

All analyses were carried out in triplicate, and results were expressed as mean ± standard deviation. The data for techno-functional and chemical parameters of the blends were evaluated using two-way analysis of variance (ANOVA) to test the effects of mango leaf cultivar and substitution level, as well as their interaction. For pasta quality parameters, one-way ANOVA was applied to compare the effects of cultivar and storage time. When significant differences were detected, Tukey’s post hoc test was performed to determine differences between means. Statistical significance was accepted at *p* < 0.05. Analyses were conducted using SPSS software (version 20, IBM Corp., Chicago, IL, USA).

## 3. Results and Discussion

### 3.1. Absorptive Profile of Mango Leaves and Chestnut Flour Blends

The absorptional characteristics of mango leaf-fortified chestnut flour blends revealed significant cultivar- and incorporation-level-dependent variations across multiple hydration and oil-binding parameters (Table 1). These functional properties are critical determinants of processing behavior and texture development in pasta manufacturing.

WHC demonstrated highly significant effects of both variety (*p* < 0.001) and variety × share interaction (*p* < 0.001), while the main effect of incorporation level (share) was non-significant. Nam Dok Mai exhibited the highest WHC at 10% incorporation (3.67 ± 0.02 g/g dm), similar to Julie (3.62 ± 0.02 g/g dm) and Irwin (3.47 ± 0.17 g/g dm), which decreased at 20%, suggesting that the polysaccharide and polyphenolic composition of these varieties impacts water retention [26]. Similar trends were reported in studies using other plant-derived powders, where higher fiber content promoted greater hydration capacity due to the presence of hydroxyl groups capable of forming hydrogen bonds with water molecules [16,25,26,27,28]. In contrast, DLO demonstrated the lowest WHC at 10% (3.20 ± 0.07 g/g dm) but increased at 20% incorporation (3.43 ± 0.09 g/g dm), representing a 7% improvement in water retention. The same behaviour was observed for Keïtt (3.40 ± 0.04 to 3.53 ± 0.03 g/g dm). This differential cultivar response to incorporation level—reflected in the significant variety × share interaction—suggests that mango leaf cultivar selection must consider the target incorporation level to optimize water retention properties. The relatively narrow range of WHC values (3.20–3.67 g/g dm) indicates that all blends possess moderate water-holding capacity, which is favorable for pasta dough formation without excessive stickiness.

WAC values showed highly significant variety and shared effects (*p* < 0.001) with no significant interaction effects. DLO demonstrated the highest WAC at both incorporation levels (2.31 ± 0.08 at 10% and 2.44 ± 0.08 at 20%), representing a 5.6% increase with higher mango leaf content, suggesting that mango leaf powder addition modified the starch–water interactions, possibly by increasing porosity. The enhancement of WAC at higher substitution levels (20%) indicates potential for improved dough hydration during pasta processing [26]. Julie and Nam Dok Mai also exhibited elevated WAC values at 20% incorporation (2.28 ± 0.07 and 2.42 ± 0.06, respectively), indicating enhanced water binding under mechanical stress conditions. Keïtt displayed the lowest WAC at 10% (2.05 ± 0.01 g/g dm), which increased substantially to 2.25 ± 0.10 at 20% (9.8% increase). IRWIN maintained a relatively stable WAC across incorporation levels (2.13 ± 0.01 to 2.26 ± 0.08). The consistently higher WAC values at 20% incorporation across most cultivars suggest that increased mango leaf content enhances the flour blend’s capacity to bind water under centrifugal force, likely due to the hydrophilic nature of mango leaf polysaccharides and proteins. This property is particularly relevant for pasta processing, where higher WAC supports better dough hydration and reduced cooking loss.

OAC measurements revealed non-significant effects for factors- variety: ns and share: ns, and significant variety × share interaction (*p* < 0.05), indicating that oil-binding properties remain relatively stable across cultivars and incorporation levels. The relatively stable OAC suggests that mango leaves powder (MLP) incorporation did not alter the protein–lipid interactions within the gluten-free matrix. Since MLP is predominantly rich in polyphenolic compounds rather than lipophilic ones, its contribution to oil-binding ability is minimal. The results align with previous observations in gluten-free formulations supplemented with plant powders low in non-polar side chains [29,30,31]. Values ranged narrowly from 1.81 ± 0.09 (Julie 20/80) to 2.14 ± 0.22 (Julie 10/90) g/g dm. Julie exhibited the highest OAC at 10% (2.14 ± 0.22 g/g dm), which decreased to 1.81 ± 0.09 a at 20%, while most other cultivars showed minimal variation. Nam Dok Mai and DLO maintained stable OAC across incorporation levels (approximately 1.91–1.96 and 1.81–1.93 g/g dm, respectively), while IRWIN and Keïtt showed slight decreasing trends with increased incorporation (1.89 to 1.88 and 1.90 to 1.93 g/g dm, respectively). The relatively uniform and moderate OAC values across all blends suggest that mango leaf addition does not substantially alter lipid-binding properties, which is advantageous for pasta manufacturing as it indicates minimal interference with fat-based ingredients or potential lipid oxidation during storage.

HLI, calculated as the ratio of WAC to OAC, showed highly significant variety effects (*p* < 0.01) and significant variety × share interaction (*p* < 0.05). Nam Dok Mai exhibited the highest HLI at both incorporation levels (1.18 ± 0.06 at 10% and 1.23 ± 0.04 at 20%), indicating a strong hydrophilic character. DLO maintained consistent HLI (1.28 ± 0.06 d at both levels), representing the most hydrophilic blend overall. In contrast, Julie demonstrated the lowest HLI at 10% (1.02 ± 0.13 g/g dm), which increased to 1.26 ± 0.03 at 20% (23.5% increase), indicating a substantial shift toward hydrophilicity with increased mango leaf content. The positive correlation between HLI and mango leaf incorporation across most cultivars suggests that mango leaves contribute predominantly hydrophilic components to the flour matrix. The increase in HLI with higher substitution levels may be attributed to the structural reorganization of the starch–fiber matrix, allowing better water penetration [32]. For pasta applications, HLI values above 1.20 (DLO, Nam Dok Mai at 20%) indicate optimal water affinity for proper dough hydration and cooking behavior.

WAI demonstrated highly significant effects for all factors (variety: *p* < 0.001, share: *p* < 0.001, variety × share: *p* < 0.001), indicating complex interactions between cultivar, incorporation level, and water absorption under thermal treatment. IRWIN exhibited the highest WAI at 10% (6.68 ± 0.25 g/g dm), which decreased markedly to 5.72 ± 0.17 at 20% (14.4% reduction). Similarly, Julie showed high WAI at 10% (6.35 ± 0.23 g/g dm) that decreased to 4.48 ± 0.76 at 20% (29.4% reduction), representing the most substantial incorporation-dependent decrease among all cultivars. In contrast, Nam Dok Mai maintained relatively stable WAI across incorporation levels (5.92 ± 0.11 to 6.06 ± 0.03), while DLO showed minimal variation (5.69 ± 0.07 to 5.62 ± 0.07). Keïtt displayed the lowest WAI at 10% (5.11 ± 0.14 g/g dm), which decreased further to 4.95 ± 0.19 at 20%. The notable decreases in WAI at 20% incorporation for some cultivars (particularly Julie and IRWIN) suggest potential matrix interference effects where higher concentrations of mango leaf components may compete with chestnut starch for water or create structures that reduce swelling capacity during heating [33].

WSI revealed highly significant variety and share effects (*p* < 0.001) with non-significant interaction. Julie exhibited the highest WSI at 10% (24.78 ± 3.60 g/g dm), followed by Keïtt (21.95 ± 4.99 g/g dm), indicating high concentrations of water-soluble components and suggesting that this formulation exhibited partial degradation of macromolecules, possibly due to the presence of enzymatically active polyphenols or enhanced dispersion of soluble components. High WSI values generally indicate greater molecular breakdown, which could influence cooking behavior and textural quality of the pasta [34]. However, both cultivars showed substantial decreases at 20% incorporation (Julie: 17.16 ± 0.78, 30.8% reduction; Keïtt: 15.62 ± 2.25, 28.8% reduction). Nam Dok Mai and DLO displayed intermediate WSI values at 10% (18.34 ± 0.39 and 18.78 ± 1.76, respectively) that decreased moderately at 20% (07.55 ± 1.44 and 15.12 ± 1.47). IRWIN showed the lowest WSI at 10% (16.34 ± 0.43 g/g dm) with a reduction to 08.44 ± 0.94 at 20% (48.3% decrease), representing the most pronounced incorporation effect. The inverse relationship between incorporation level and WSI across all cultivars suggests that higher mango leaf concentrations may promote entrapment of soluble components within insoluble matrices during thermal treatment, or that interactions between mango leaf polyphenols and proteins/polysaccharides reduce extractability. For pasta applications, moderate WSI values (15–18 g/g dm) are optimal to minimise cooking losses while maintaining structural integrity.

SP measurements showed non-significant effects for all factors (variety: ns, share: ns, variety × share: ns), though numerical trends were apparent. This suggests that starch–starch interactions were not markedly disrupted by MLP addition, possibly due to its limited starch content and predominant fibrous nature. The retention of similar SP values across treatments may be advantageous for maintaining pasta texture during cooking [35]. Julie maintained the highest SP at 10% (6.84 ± 0.31 g/g dm) that decreased to 5.49 ± 1.69 at 20%, while IRWIN showed elevated SP (6.85 ± 0.36 at 10% and 6.50 ± 0.52 at 20%). Nam Dok Mai and DLO demonstrated relatively stable SP across incorporation levels (6.48 to 6.53 and 6.05 to 5.96 g/g dm, respectively). Keïtt displayed moderate SP values (5.94 ± 0.69 to 5.45 ± 0.63), showing a slight decreasing trend with increased incorporation. The uniform SP values across samples (ranging from 4.48 to 6.85 g/g dm) indicate that all blends possess adequate swelling capacity for pasta applications, ensuring proper starch gelatinization during cooking. The non-significant statistical effects suggest that SP is less sensitive to mango leaf cultivar and incorporation level than other absorptional parameters, providing processing consistency across formulations.

### 3.2. Total Polyphenols Content and Antioxidant Capacity of Mango Leaves and Chestnut Flour Blends

**Figure 1:**
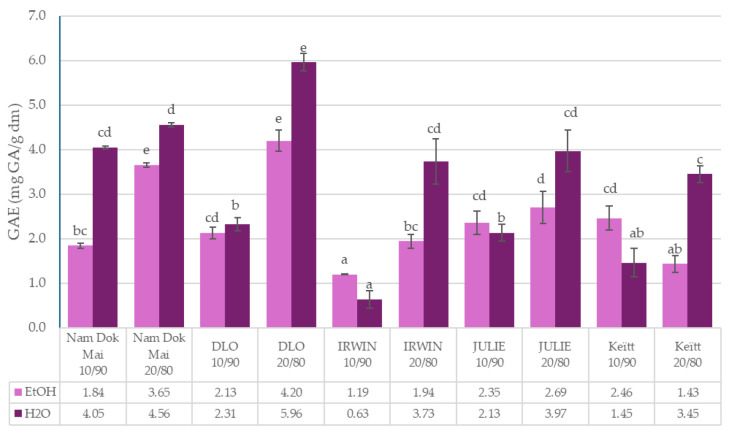
Total polyphenol content (TPC) of mango leaves and chestnut flour blends. Lowercase letters mean significant differences in columns at *p* = 0.05.

**Figure 2:**
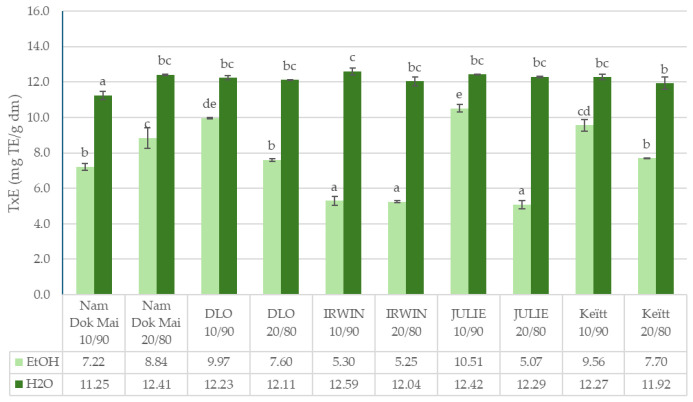
Antioxidant activity (DPPH) of mango leaves and chestnut flour blends. Lowercase letters mean significant differences in columns at *p* = 0.05.

**Figure 3:**
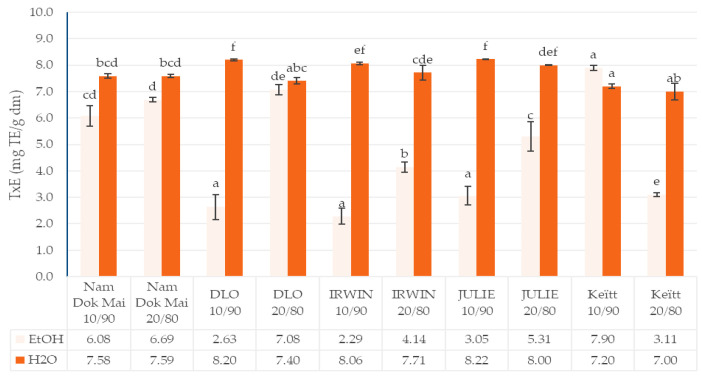
Antioxidant capacity (ABTS) of mango leaves and chestnut flour blends. Lowercase letters mean significant differences in columns at *p* = 0.05.

**Figure 4:**
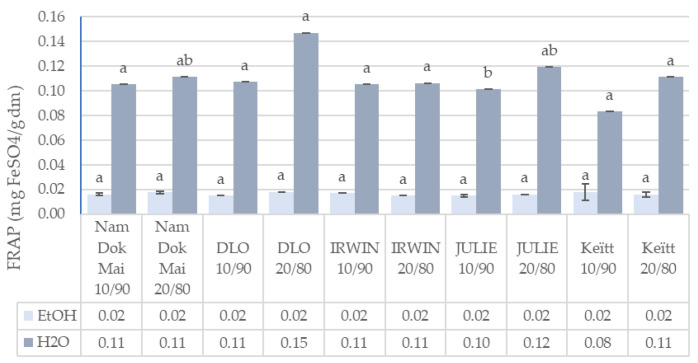
Ferric reducing antioxidant power (FRAP) of mango leaves and chestnut flour blends. Lowercase letters mean significant differences in columns at *p* = 0.05.

The data presented in Figure 1, Figure 2, Figure 3 and Figure 4 reveal significant cultivar-dependent variations in total phenolic content and antioxidant capacity as influenced by mango leaf powder incorporation level (10% vs. 20% addition to chestnut flour) and extraction solvent system (ethanol vs. water).

For TPC measured in ethanol extracts, Nam Dok Mai exhibited the highest values at both incorporation levels (3.65 ± 0.05 at 20% and 1.84 ± 0.06 at 10%), demonstrating a clear dose-dependent response. IRWIN showed the lowest TPC in ethanol extracts at 10% incorporation (1.19 ± 0.01 a). TPC values measured in water extracts displayed a different pattern, with DLO displaying the highest phenolic content at 20% incorporation (5.96 ± 0.2), while Keïtt showed relatively lower values at 10% (1.45 ± 0.32). Notably, all cultivars demonstrated approximately 1.5–2.5× increased TPC values at 20% incorporation compared to 10%, confirming a proportional relationship between mango leaf addition and extractable phenolic content. Interestingly, TPC in water extracts was generally comparable to or higher than TPC in ethanol extracts across most samples, particularly evident in DLO 20/90 (5.96 ± 0.2 vs. 4.20 ± 0.23) and Nam Dok Mai 20/90 (4.55 ± 0.05 vs. 3.65 ± 0.05). This dose-dependent effect was highly significant (*p* < 0.001), indicating that higher mango leaf incorporation levels effectively enrich chestnut flour blends with phenolic compounds, and that water extraction is comparable or more efficient than ethanol for recovering total phenolics from these matrices.

Studies on young leaves of various mango (*Mangifera indica* L.) varieties grown in the Chiang Mai region of Thailand also showed that the Nam Dok Mai variety has a high content of phenolic compounds, mainly in the form of substances such as maclurin glucoside (28.73 mg/g dw), iriflophenone glucoside (iriflophenone 3-C-β-D-glucopyranoside) (23.34 mg/g dw), mangiferin (33.27 mg/g dw), mangiferin pentoside (14.20 mg/g dw), and quercetin hexoside (6.41 mg/g dw) [36]. Iriflophenone glucoside and maclurin glucoside are polyphenols that are responsible for the health-promoting properties of young mango leaves, primarily their antidiabetic properties. These properties are related to the confirmed inhibitory activity of iriflofenone-3-glucoside against α-glucosidase, which means that raw materials and food products enriched with this polyphenol can be used in the treatment of type 2 diabetes, which is associated with a high risk of developing atherosclerosis [37].

In addition to its antidiabetic effects, mangiferin, present in Nam Dok Mai mango leaves, is believed to have additional health-promoting properties, including anti-inflammatory, anticancer, cardioprotective, and neuroprotective effects, as well as a role in limiting the development of conditions such as hyperuricemia and obesity [38]. Mangiferin may also protect hepatocytes from damage caused by free oxygen radicals and oxidative substances, for example, by forming mangiferin:Fe^3+^ complexes [38]. For this reason, young Nam Dok Mai mango leaves, whose phenolic compounds are efficiently extracted in both water and alcohol, may be a potential raw material for functional foods for diabetics [37].

The DPPH assay results revealed marked differences between extraction systems and incorporation levels. For DPPH activity in ethanol extracts, Julie and DLO demonstrated superior antioxidant capacity at 10% incorporation (10.51 ± 0.22 and 9.97 ± 0.03, respectively), while DLO maintained the highest activity at 20% (7.60 ± 0.09). However, DPPH activity in water extracts was substantially higher across all samples, with all cultivars exhibiting values above 11 mg TE/g DM. The highest scavenging activities were observed in Nam Dok Mai at 20% incorporation (12.40 ± 0.04) and Julie at 10% incorporation (12.42 ± 0.01). The solvent effect on DPPH activity was highly significant (*p* < 0.001), with water extracts consistently demonstrating 1.2–1.9× higher radical scavenging capacity compared to ethanol extracts. Interestingly, the pattern of incorporation-level response differed between solvents: ethanol extracts generally showed decreased DPPH activity at 20% incorporation (suggesting potential matrix interference effects), while water extracts maintained consistently high activity across both incorporation levels. This suggests that water extraction is more effective at recovering dose-independent antioxidant compounds, likely including highly polar phenolics such as mangiferin and gallic acid derivatives, whose scavenging capacity is not proportionally affected by the flour matrix concentration.

It was also confirmed that mango leaves contain significant amounts of ascorbic acid [14], which could also be dissolved to a certain extent in our extraction using aqueous-ethanol solutions (90/10 ethanol/water mixtures). This observation may be confirmed by the results obtained for the Julie and DLO cultivars using water extraction (with 10% incorporation), where higher antioxidant activity was observed compared to ethanol extraction, which may be due to the intense dissolution of highly polar antioxidants, such as ascorbic acid, in water. Our results partially confirm studies that found a higher ascorbic acid content in the Julie cultivar fruit (55.00 mg/100 g d.b.), compared to the Keith cultivar (47.33 mg/100 g d.b.) [39,40].

The higher antioxidant activity demonstrated for the DLO, Julie, and Keitt varieties with a 10% mango leaf powder content, and lower with a 20% mango leaf powder content, may likely result from the antioxidants from the leaves of these varieties reaching their maximum solubility concentration in the solvent used, ethanol. On the other hand, increasing the amount of bioactive material to 20% could have resulted in the extraction of increased amounts of antioxidant substances, which lost their antioxidant activity at high concentrations, as a nonlinear relationship between antioxidant activity and the content of antioxidant substances in the extract was confirmed [41]. At higher antioxidant concentrations (obtained from 20% of the bioactive raw material), some antioxidant substances (e.g., some polyphenols) change their activity from antioxidant to pro-oxidant, which was observed for ferulic acid and cyanidin-3-O-glucoside exhibited significantly higher antioxidant activity at a concentration of 5 µmol/l than at a concentration of 50 µmol/l, while 3,4-dihydroxyphenyl-acetic acid showed no differences in antioxidant activity when present in solution at various concentrations ranging from 5 µmol/l to 50 µmol/l [41].

The significantly higher antioxidant activity confirmed in all analysed samples in aqueous extracts compared to ethanol extracts reflects the differences in the polarity of the solvents used and the chemical components present in the leaves of the studied mango varieties, specifically mangiferin. Most of the mangiferin in mango leaves occurs as a glycoside, in the form of 1,36,7-tetrahydroxyxanthone-C2-β-D-glucoside, in which the xanthone moiety is linked to a glucose molecule [42], which makes the mangiferin molecule highly polar and therefore highly water-soluble. Therefore, water can be considered a more effective solvent for antioxidant substances contained in mango leaves than an ethanol-water mixture.

ABTS activity measurements also showed consistently higher values in water extracts compared to ethanol extracts across all cultivars and incorporation levels. At 10% incorporation, Julie, IRWIN, and Keïtt exhibited relatively higher ABTS activity in water extracts (8.22 ± 0.00, 8.06 ± 0.04, and 7.20 ± 0.08, respectively), while Nam Dok Mai and DLO demonstrated moderate ABTS activity in ethanol extracts (6.69 ± 0.08 and 7.08 ± 0.19) at 20% incorporation. The data indicates a strong solvent-dependent response (*p* < 0.001), with ABTS values in water extracts being approximately 15–60% higher than corresponding measurements in ethanol extracts. The most pronounced solvent effect was observed in IRWIN 10/90 (8.06 ± 0.04 ef for water vs. 2.29 ± 0.30 for ethanol), representing a 3.5-fold increase in scavenging capacity with water extraction. Furthermore, increasing mango leaf incorporation from 10% to 20% showed variable responses depending on cultivar and solvent: ethanol extracts generally showed modest increases (except for Julie), while water extracts exhibited more stable or slightly decreased values. This suggests that different classes of antioxidant compounds—potentially those with varying polarities and molecular structures—contribute differentially to ABTS scavenging mechanisms, and that water-soluble antioxidants may approach saturation levels at 10% incorporation.

The ABTS method also demonstrated high activity in aqueous extracts for the DLO and Julia varieties, which may suggest that the antioxidants present in these varieties (such as ascorbic acid and mangiferin glucoside (1,36,7-tetrahydroxyxanthone-C2-β-D-glucoside)) neutralize the ABTS•+ cation radical with similarly strong antioxidant activity. However, the opposite observation compared to DPPH (except for the Keitt variety) regarding an increase in antioxidant activity with increasing amounts of extracted bioactive components may be due to the fact that antioxidants other than those susceptible to conversion into pro-oxidants at high concentrations in the sample were involved in the reaction with the ABTS•+ cation radical [41].

The analysis conducted using the ABTS assay confirmed that, in terms of antioxidant activity, Dlo mango leaves can be a beneficial raw material for the production of functional foods.

FRAP values demonstrated relatively modest variation among cultivars and incorporation levels, with measurements in ethanol extracts falling within a narrow range (0.01–0.02 mg FeSO_4_/g DM) and values in water extracts ranging from 0.08 to 0.15 mg FeSO_4_/g DM. Nam Dok Mai showed consistent FRAP in ethanol extracts across incorporation levels (0.02 − 0.018 ± 0.00), while no significant differences were observed for FRAP in water extracts at 10% across most cultivars (0.11 ± 0.01 uniformly for Nam Dok Mai, IRWIN, and Julie). At 20% incorporation, DLO displayed the highest FRAP in water extracts (0.15 ± 0.02), followed by Julie (0.12 ± 0.01) and Keïtt (0.11 ± 0.00). The solvent effect on FRAP was highly significant (*p* < 0.001), with water extracts showing approximately 6–9× higher reducing power compared to ethanol extracts. However, the relatively uniform FRAP values in ethanol extracts across samples, combined with the significant cultivar effect (*p* < 0.05) for FRAP in water extracts, suggest that water-soluble reducing compounds show greater cultivar-dependent variation than ethanol-soluble counterparts.

The observed high reduction potential (at 20% incorporation) for DLO mango leaves in the aqueous extract may indicate that this variety likely contains a large amount of reducing compounds responsible for the reduction of Fe^3+^ ions to Fe^2+^, such as ascorbic acid and phenolic acids such as gallic acid, protocatechuic acid, caffeic acid, and chlorogenic acid [43,44]. In the case of the FRAP method used (which is based on the electron transfer mechanism), the high activity of aqueous extracts may therefore confirm the effective extraction of compounds with a low redox potential (i.e., high reduction potential), such as ascorbic acid [45,46].

The observed, cultivar-dependent variability in reducing capacity in an aqueous environment may indicate that the total reducing capacity of the analyzed mixtures depends more on polar antioxidants and secondary metabolites than on nonpolar antioxidants and secondary metabolites present in the leaves of individual mango cultivars. Our results indicate that the leaves of the DLO and Julie cultivars may be valuable raw materials in food production and technology.

### 3.3. Reducing Sugar Content in Mango Leaves and Chestnut Flour Blends

**Figure 5:**
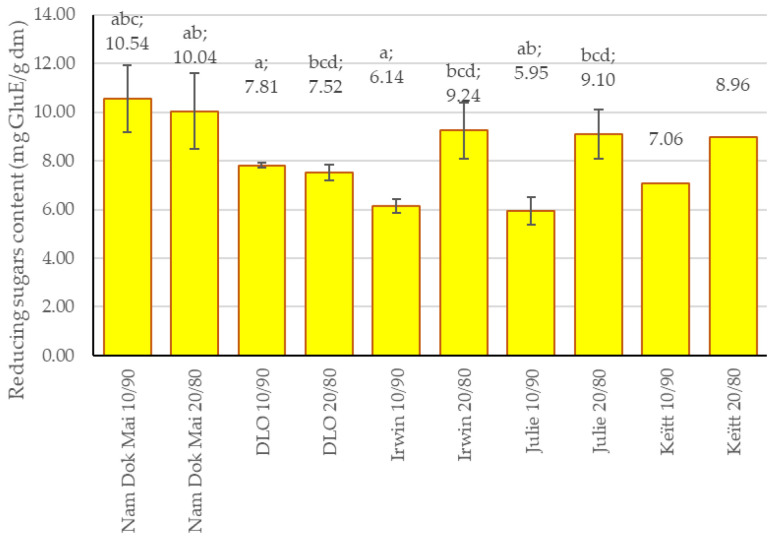
Reducing the sugar content of mango leaves and chestnut flour blends. Lowercase letters mean significant differences in columns at *p* = 0.05.

The reducing sugar content analysis revealed significant cultivar- and incorporation-level-dependent variations across the mango leaf-fortified chestnut flour blends (Figure 5).

Nam Dok Mai demonstrated the highest reducing sugar content at 10% incorporation (10.54 mg GluE/g DM, abc), followed closely by the 20% incorporation level (10.04 mg GluE/g DM, ab). Julie and Keïtt at 20% incorporation also exhibited elevated reducing sugar levels (9.10 mg GluE/g DM, bcd and 8.96 mg GluE/g DM, respectively), along with Irwin at 20% (9.24 mg GluE/g DM, bcd).

Interestingly, the incorporation-level effect on reducing sugars varied markedly by cultivar. While Nam Dok Mai showed relatively stable reducing sugar content across both incorporation levels (10.54 vs. 10.04 mg GluE/g DM), other cultivars demonstrated increases at 20% incorporation: Irwin increased from 6.14 (a) to 9.24 mg GluE/g DM (bcd), and Julie increased from 5.95 (ab) to 9.10 mg GluE/g DM (bcd). This 1.5-fold increase suggests that higher mango leaf incorporation releases or makes available additional reducing sugars, potentially through enzymatic or chemical breakdown during processing, or through increased extraction efficiency from the higher concentration of leaf material. DLO exhibited moderate and stable reducing sugar content at both incorporation levels (7.81 mg GluE/g DM, and at 10% and 7.52 mg GluE/g DM, bcd at 20%), showing minimal variation and falling in the mid-range across all samples. The lowest reducing sugar contents were observed in Irwin 10/90 (6.14 mg GluE/g DM, a) and Julie 10/90 (5.95 mg GluE/g DM, ab), while Keïtt 10/90 showed intermediate values (7.06 mg GluE/g DM).

The high content of reducing sugars in the Nam Dok Mai cultivar, which was largely independent of the degree of leaf powder incorporation, may indicate an intensive process of soluble carbohydrate accumulation. Research results indicate that in most plant species, the vast majority of soluble sugars occur in the form of glucose, fructose, and sucrose [47]. These substances are likely responsible for the high total reducing sugar content in the Nam Dok Mai cultivar, which can serve as both an energy source and a substrate for polysaccharide synthesis in the mango plant [48]. It is important to note that reducing sugars, such as glucose and fructose, are not only primary metabolites but can also play a significant role in plant adaptation to environmental stresses. It has been confirmed that their increased concentration can increase plant tolerance (by influencing, among other things, cell osmotic pressure) to abiotic stressors such as low water availability, high soil salinity, and low temperatures [49].

Therefore, our results suggest that Nam Dok Mai mango leaves, by regulating carbohydrate levels, may be particularly adapted to the growing environment not only in the plant’s place of origin (Thailand) but also in other, less favourable agricultural regions of the world, such as Egypt, Pakistan, and Nigeria [50].

Based on the results of functional and compositional analyses, the following blends were selected for rheological evaluation: CF (chestnut flour control) and mango leaf-chestnut flour blends with mixing ratios of Nam Dok Mai 10/90, Nam Dok Mai 20/80, Irwin 10/90, Irwin 20/80, Julie 10/90, and Julie 20/80. These cultivars were selected based on the following criteria: (1) superior antioxidant activity across multiple assays (DPPH, ABTS, FRAP), particularly Irwin, Nam Dok Mai, and Julie; (2) favorable water absorption and hydration properties (WAI, WHC) essential for pasta dough formation; and (3) diverse functional profiles representing different absorption and solubility behaviors. DLO and Keitt, despite showing interesting properties, exhibited either lower overall antioxidant activity (DLO) or very high WSI values (Keitt) that might compromise pasta structural integrity during cooking, and were therefore excluded from pasta production trials.

### 3.4. Viscometric Properties of Chosen Mango Leaves and Chestnut Flour Blends

**Figure 6:**
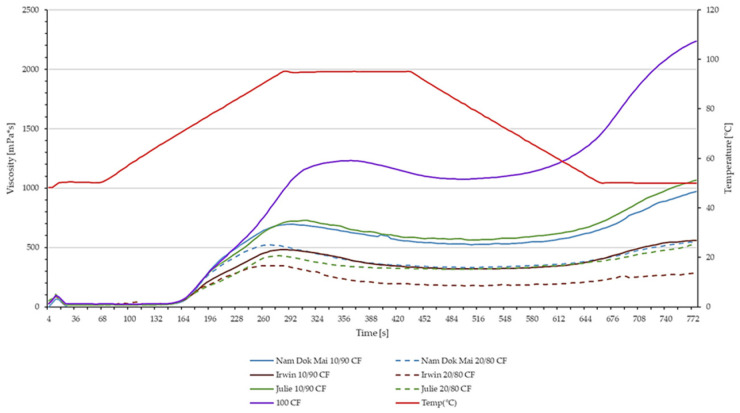
Pasting profile of mango leaves and chestnut flour blends.

The pasting behavior of starch-based foods provides valuable insight into the functional and textural characteristics that govern product quality during processing and storage (Figure 6, Table 2).

The pasting behavior of starch-based foods provides valuable insight into the functional and textural characteristics that govern product quality during processing and storage. Pure chestnut flour exhibited a peak viscosity of 1233.5 ± 55.5 mPa·s, while all mango leaf blends showed substantially lower values. Nam Dok Mai blends ranged from 521.5–698.0 mPa·s, Irwin blends from 350.0–481.5 mPa·s, and Julie blends from 431.0–732.5 mPa·s, representing 28–74% reductions compared to pure chestnut flour. The decline in peak viscosity with increasing levels of MLP indicates a dilution effect caused by partial replacement of starch with non-starch components, particularly dietary fiber and phenolic compounds. Similar findings were reported by Santamaria et al. (2025) [51] and Delgado-Ospina et al. (2021) [52], who observed that high-fiber and polyphenol-rich plant powders reduced starch swelling capacity due to limited water availability and physical hindrance of granule expansion. The hydroxyl groups present in fibers and phenolics can strongly bind water molecules through hydrogen bonding, thus competing with starch for hydration and reducing paste viscosity. Moreover, the phenolic–amylose interactions may limit granule disintegration and restrict amylose leaching, leading to lower viscosity development during heating [53,54,55]. The most notable impact was observed in final viscosity, where pure chestnut flour reached 2237.0 ± 63.0 mPa·s, while mango leaf additions reduced this to 277.0–1066.0 mPa·s. The setback values, indicating retrogradation tendency, decreased from 1161.5 mPa·s (pure CF) to 100.5–502.0 mPa·s in the blends, suggesting reduced gel-forming capacity. Julie 10/90 CF demonstrated the best performance among blends, achieving 732.5 mPa·s peak viscosity and 1066.0 mPa·s final viscosity. Nam Dok Mai varieties showed concentration-dependent behaviour (10/90 > 20/80), while Irwin varieties exhibited the lowest viscosities, particularly at higher concentrations.

Breakdown viscosity, which reflects the paste’s stability under shear and heat, was generally lower in MLP-enriched samples, remained relatively consistent (115.0–189.5 mPa·s) across all formulations compared to pure chestnut flour (158.0 mPa·s), indicating improved thermal stability of the starch matrix. This effect may result from the reinforcement of the paste structure by fiber–polyphenol–starch interactions, forming a more cohesive network that resists granule collapse [32,35]. Although the total viscosity was reduced, the lower breakdown suggests a more stable paste, which is advantageous for processing gluten-free systems prone to excessive viscosity changes during cooking.

Pasting temperatures showed minimal variation (65.97–73.08 °C), suggesting mango leaf addition does not significantly affect gelatinization onset. Although Irwin 20/80 exhibited a notably lower value, which could be attributed to differences in starch–polyphenol interactions or reduced starch crystallinity due to high substitution levels. These variety-dependent differences reflect the heterogeneity in the chemical composition of mango leaves—particularly variations in fibre type, phenolic concentration, and ash content—which can differently modulate starch gelatinisation behaviour. The results indicate that mango leaf flour incorporation reduces starch functionality, with variety and concentration being critical factors in maintaining acceptable pasting properties for food applications.

Based on the pasting parameters, Nam Dok Mai and Julie were selected for spaghetti manufacturing trials due to their superior viscosity profiles and structural stability compared to Irwin. Nam Dok Mai at lower incorporation demonstrated the highest peak viscosity, trough viscosity, and final viscosity among all mango-fortified blends, indicating excellent starch functionality and structural integrity essential for pasta texture development. Julie at lower incorporation exhibited the second-highest peak viscosity but achieved the highest final viscosity among all mango-fortified blends and the highest setback value, suggesting superior starch retrogradation capacity critical for maintaining firm texture after cooking and cooling, a key quality attribute for pasta al dente characteristics. In contrast, Irwin demonstrated substantially lower viscosity values across all pasting parameters, with peak viscosity declining visibly at higher incorporation levels, along with marked reductions in final viscosity and setback values. These significantly reduced pasting parameters indicate weakened starch gelatinisation and poor retrogradation behaviour that would likely result in soft, structurally compromised pasta with inadequate firmness. Additionally, Irwin at higher incorporation showed an unusually low and highly variable pasting temperature, suggesting processing instability and unpredictable thermal behavior.

Both Nam Dok Mai and Julie maintained pasting temperatures similar to the chestnut flour control, ensuring predictable and consistent processing behavior compatible with standard pasta manufacturing protocols. The combination of adequate viscosity development, strong retrogradation potential reflected in substantial setback values, and stable thermal pasting characteristics positioned Nam Dok Mai and Julie as optimal choices for producing spaghetti with acceptable texture, firmness, and cooking quality.

### 3.5. Quality Features of Spaghetti Made from Chosen Mango Leaves Blends

#### 3.5.1. Color Changes in Spaghetti During Processing and Storage

**Figure 7:**
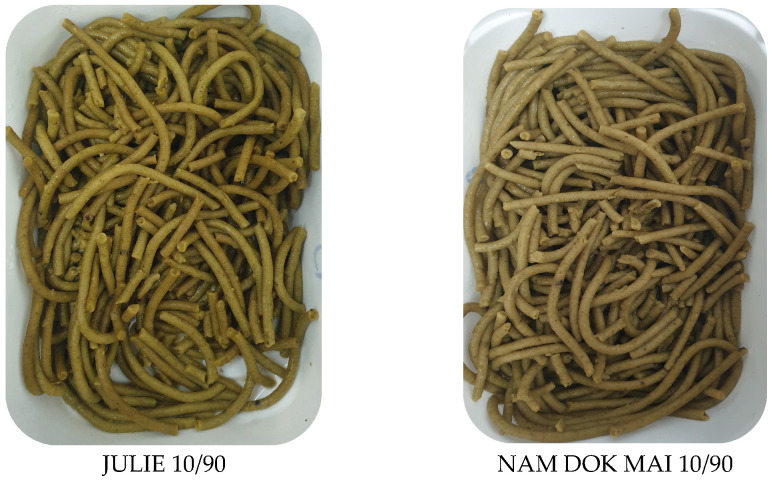
Spaghetti prepared from JULIE 10/90 and NAM DOK MAI 10/90 blends.

**Figure 8:**
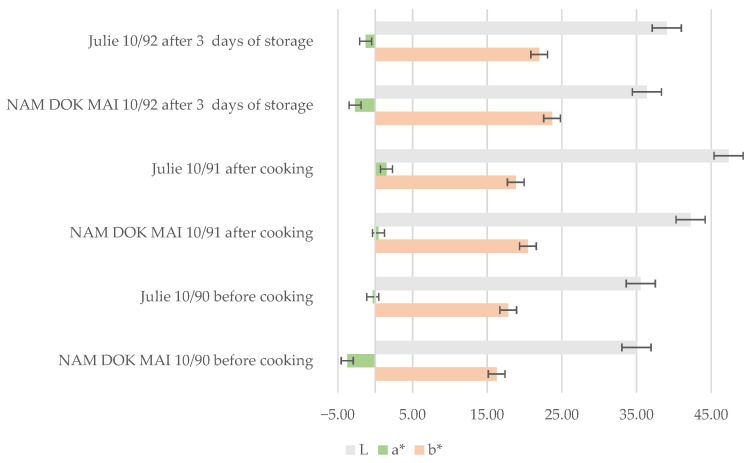
Color changes in spaghetti before and after cooking, and after 3 days of storage.

Before cooking, both formulations exhibited similar color profiles with moderate lightness values and comparable yellowness characteristic of mango leaf-fortified pasta (Figure 7 and Figure 8).

The a* parameter indicated slight greenish hues in both formulations, consistent with chlorophyll and other pigments from mango leaf incorporation. Initial color uniformity suggested that raw pasta appearance was not substantially influenced by cultivar at the incorporation level tested. The observed color differences between the Nam Dok Mai and Julie spaghetti formulations during processing and storage can be attributed to cultivar-specific pigment stability and oxidative resistance. The better lightness and yellowness retention in Nam Dok Mai aligns with previous findings on vegetable-enriched pasta, where certain plant matrices preserved carotenoids and flavonoids more effectively during thermal treatment and storage [56].

After cooking, the cultivars demonstrated markedly divergent color responses. Nam Dok Mai spaghetti showed increased lightness following cooking, indicating favorable color retention during hydration and thermal treatment. The yellowness parameter increased moderately while greenish tone was largely maintained, resulting in an appealing golden-green appearance. Julie spaghetti exhibited substantial lightness reduction after cooking, indicating significant Maillard reaction activity, oxidation of phenolic compounds, or both during cooking. This darkening would be visually problematic for pasta products, though moderate yellowness values were maintained [57,58].

The three-day storage period revealed further cultivar-dependent color stability differences. Nam Dok Mai maintained relatively stable lightness compared to the freshly cooked state, demonstrating good color retention and resistance to oxidative browning. The yellowness parameter showed slight increases with minimal but acceptable color changes, while the greenish hue remained detectable. Julie continued to exhibit substantially reduced lightness after storage with no recovery from the cooking-induced darkening [59].

#### 3.5.2. Cutting Force of Spaghetti

The cutting force measurements showed no significant differences between Nam Dok Mai and Julie spaghetti (Julie 10/90 − 3.64 ±0.66 N and NAM DOK MAI 10/91 3.11 ±0.42 N), indicating comparable textural firmness despite their different pasting profiles. Despite Julie’s superior setback viscosity, no measurable difference in cutting force was detected, reinforcing evidence that starch retrogradation properties do not always translate into firmer textures after cooking, particularly in the presence of plant-based fibers. Both formulations achieved acceptable mechanical properties suitable for pasta applications. However, Nam Dok Mai demonstrated superior performance in maintaining desirable color and texture attributes after cooking and storage, making it the more promising cultivar for pasta fortification applications [60].

#### 3.5.3. Antioxidant Activity of Spaghetti

**Figure 9:**
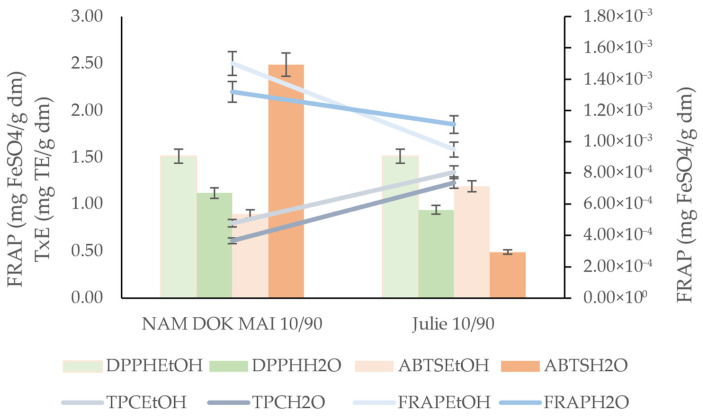
Total polyphenol and antioxidant characteristics of spaghetti.

The total polyphenol content and antioxidant activities of cooked spaghetti revealed cultivar-dependent retention of bioactive compounds following pasta processing and cooking (Figure 9).

Total phenolic content measured in ethanol extracts showed Nam Dok Mai maintained higher values than Julie, with water extracts following a similar pattern. This indicates that Nam Dok Mai’s phenolic compounds withstood the combined stresses of pasta extrusion, drying, and boiling more effectively than Julie’s phenolics. The preservation of TPC in both extraction systems suggests that both hydrophobic and hydrophilic phenolic fractions remained bioavailable in the final cooked product.

Analysis of the total polyphenol content (TPC) in the resulting product (spaghetti) suggests that the polyphenols in the leaves of the Nam Dok Mai variety may have a more stable chemical structure compared to those found in the Julie variety. It has been demonstrated that the stability of polyphenols is significantly dependent on the degree of hydroxylation, acylation, and glycosylation, among other factors. Highly hydroxylated polyphenols exhibit reduced thermal stability, while high levels of glycosylation increase the polyphenols’ resistance to high temperatures [61]. It has also been confirmed that acylated polyphenols are more resistant to elevated temperatures than non-acylated polyphenols [62]. Therefore, it can be assumed that due to the potentially greater stability of the bioactive components in the Nam Dok Mai variety, this raw material is less susceptible to the degradation of polyphenolic substances during thermal processing.

DPPH radical scavenging activity in cooked spaghetti exhibited the same cultivar preference, with Nam Dok Mai showing elevated values in both ethanol and water extracts compared to Julie. The retention of DPPH activity in water extracts is particularly relevant for nutritional applications, as these represent the readily accessible antioxidants available during consumption. The lower DPPH values in Julie spaghetti correlate with the substantial colour darkening observed during cooking (Figure 7), suggesting that oxidation and Maillard reactions consumed reactive phenolic compounds, reducing their radical scavenging capacity. It is possible that the phenolic fraction in Julie spaghetti is less strongly bound to cell wall matrices and therefore more easily oxidized [63], leading to the inactivation of polyphenols and, consequently, a reduction in the antioxidant activity of the cooked pasta.

ABTS activity measurements confirmed the antioxidant retention in Nam Dok Mai, particularly in water extracts, where the difference was most pronounced. The elevated ABTS values in Nam Dok Mai indicate preservation of water-soluble antioxidants, including mangiferin and other polar phenolic compounds that survived thermal processing. Julie showed reduced ABTS activity, especially in water extracts, consistent with the degradation or transformation of water-soluble antioxidants during cooking.

The results obtained regarding higher antioxidant activity (measured by the ABTS method) confirmed that, in the case of Nam Dok Mai spaghetti, mainly polar antioxidants were effectively extracted. At the same time, it can be assumed that the bioactive substances (e.g., polyphenols) in Nam Dok Mai spaghetti, which retain high antioxidant activity despite thermal processing, are less thermally labile and less susceptible to browning reactions than in Julie spaghetti. As a result, the individual phenolic molecules present in Nam Dok Mai spaghetti may polymerise less readily into large, colourful polymer molecules [64], which is reflected in the high antioxidant activity (ABTS, in aqueous extracts) despite thermal processing.

Ferric reducing antioxidant power analysis revealed Nam Dok Mai maintains higher reducing capacity in both solvent systems compared to Julie. The results of analyses performed using FRAP, as well as other methods (DPPH, ABTS, and Folin–Ciocalteu reagent), can confirm the overall high health-promoting value of the finished product (spaghetti).

## 4. Conclusions

Incorporation of mango leaf powder into gluten-free pasta formulations modified the rheological and functional behavior of chestnut flour by reducing overall starch viscosity while maintaining acceptable pasting stability and thermal consistency. Among the mango cultivars tested, Nam Dok Mai and Julie exhibited the most suitable profiles for pasta processing. Although both produced spaghetti with comparable firmness, Nam Dok Mai demonstrated better color preservation, oxidative stability, and antioxidant retention during cooking and storage. The superior functional behavior of Nam Dok Mai is likely associated with more thermally stable polyphenols, as evidenced by retained TPC and antioxidant activity after thermal processing. The specific compositional factors responsible for these differences—potentially including fiber type and content, polyphenol structural characteristics, or other phytochemical profiles—require further investigation through detailed compositional analysis.

## Figures and Tables

**Table 1:** The absorption characteristics of mango leaves and chestnut flour blends.

Sample	WHC	WAC	OAC	HLI	WAI	WSI	SP
	(g H_2_O/g dm)	(g H_2_O/g dm)	(g Oil/g dm)	(g H_2_O/g dm)	(g H_2_O/g dm)	(g/g dm)	(g H_2_O/g dm)
**Nam Dok Mai 10/90**	3.67 ± 0.02 f	2.25 ± 0.09 bcd	1.91 ± 0.02 a	1.18 ± 0.06 bcd	5.92 ± 0.11 cd	18.34 ± 0.39 a	6.48 ± 0.12 ab
**Nam Dok Mai 20/80**	3.55 ± 0.02 def	2.42 ± 0.06 ef	1.96 ± 0.06 ab	1.23 ± 0.04 cd	6.06 ± 0.03 cd	07.55 ± 1.44 bc	6.53 ± 0.23 ab
**DLO 10/90**	3.20 ± 0.07 a	2.31 ± 0.08 de	1.81 ± 0.15 a	1.28 ± 0.06 d	5.69 ± 0.07 c	18.78 ± 1.76 bc	6.05 ± 0.37 ab
**DLO 20/80**	3.43 ± 0.09 cd	2.44 ± 0.08 f	1.93 ± 0.18 ab	1.28 ± 0.06 d	5.62 ± 0.07 c	15.12 ± 1.47 b	5.96 ± 0.36 ab
**IRWIN 10/90**	3.47 ± 0.17 cd	2.13 ± 0.01 ab	1.89 ± 0.13 a	1.13 ± 0.08 abc	6.68 ± 0.25 e	16.34 ± 0.43 b	6.85 ± 0.36 b
**IRWIN 20/80**	3.29 ± 0.09 ab	2.26 ± 0.08 cd	1.88 ± 0.06 a	1.20 ± 0.03 cd	5.72 ± 0.17 c	08.44 ± 0.94 a	6.50 ± 0.52 ab
**JULIE 10/90**	3.62 ± 0.02 ef	2.16 ± 0.05 abc	2.14 ± 0.22 b	1.02 ± 0.13 a	6.35 ± 0.23 de	24.78 ± 3.60 d	6.84 ± 0.31 b
**JULIE 20/80**	3.43 ± 0.06 cd	2.28 ± 0.07 cd	1.81 ± 0.09 a	1.26 ± 0.03 d	4.48 ± 0.76 a	17.16 ± 0.78 b	5.49 ± 1.69 a
**Keitt 10/90**	3.40 ± 0.04 bc	2.05 ± 0.01 a	1.90 ± 0.06 a	1.08 ± 0.04 ab	5.11 ± 0.14 b	21.95 ± 4.99 cd	5.94 ± 0.69 ab
**Keitt 20/80**	3.53 ± 0.03 cde	2.25 ± 0.10 bcd	1.93 ± 0.07 a	1.17 ± 0.05 bcd	4.95 ± 0.19 ab	15.62 ± 2.25 b	5.45 ± 0.63 a
variety	***	***	ns	**	***	***	ns
share	ns	***	ns	**	***	***	ns
varietyxshare	***	ns	*	*	***	ns	ns

WHC—water-holding capacity; WAC—water absorption capacity; OAC—oil absorption capacity; HLI—hydrophilic–lipophilic index; WAI—water absorption index; WSI—water solubility index; SP—swelling power; dm—dry matter. Lowercase letters mean significant differences in columns at *p* = 0.05, *—statistically different at *p* < 0.05; **—statistically different at *p* < 0.01; ***—statistically different at *p* < 0.001; ns—statistically non-different.

**Table 2:** Pasting parameters of mango leaves and chestnut flour blends.

Sample	Peak Viscosity (mPa·s)	Trough Viscosity (mPa·s)	Breakdown (mPa·s)	Final Viscosity (mPa·s)	Setback (mPa·s)	Pasting Time (s)	Pasting Temperature (°C)
CF	1233.5 ± 55.5 ^e^	1075.5 ± 46.5	158.0 ± 9.0	2237.0 ± 63.0	1161.5 ± 16.5	6.00 ± 0.13	71.88 ± 0.03
Nam Dok Mai 10/90	698.0 ± 6.0 ^d^	524.5 ± 7.5	173.5 ± 1.5	970.0 ± 21.0	445.5 ± 13.5	4.87 ± 0.06	72.10 ± 0.25
Nam Dok Mai 20/80	521.5 ± 18.5 ^c^	332.0 ± 6.0	189.5 ± 12.5	546.5 ± 5.5	214.5 ± 0.5	4.46 ± 0.06	71.72 ± 0.02
Irwin 10/90	481.5 ± 0.5 ^c^	318.5 ± 2.5	163.0 ± 2.0	559.0 ± 8.0	240.5 ± 5.5	4.70 ± 0.03	72.70 ± 0.00
Irwin 20/80	350.0 ± 1.0 ^a^	176.5 ± 2.5	173.5 ± 3.5	277.0 ± 5.0	100.5 ± 7.5	4.50 ± 0.10	65.97 ± 7.57
Julie 10/90	732.5 ± 2.5 ^d^	564.0 ± 1.0	168.5 ± 3.5	1066.0 ± 29.0	502.0 ± 28.0	5.04 ± 0.17	71.82 ± 0.02
Julie 20/80	431.0 ± 27.0 ^b^	316.0 ± 20.0	115.0 ± 7.0	525.5 ± 51.5	209.5 ± 31.5	4.60 ± 0.07	73.08 ± 0.38

Lowercase letters mean significant differences in columns at *p* = 0.05, CF—chestnut flour.

## Data Availability

The original contributions presented in this study are included in the article. Further inquiries can be directed to the corresponding author.
